# The Spousal Unit and Dementia: Investigating the Relational Basis of the Couplehood Concept

**DOI:** 10.3390/healthcare11152191

**Published:** 2023-08-03

**Authors:** Bernhard Weicht, Edward Tolhurst

**Affiliations:** 1 Department of Sociology, University of Innsbruck, 6020 Innsbruck, Austria; 2 School of Health, Science and Wellbeing, Staffordshire University, Stoke-on-Trent ST4 2DE, UK; e.tolhurst@staffs.ac.uk

**Keywords:** care, couplehood, dementia, personhood, relational sociology, spousal relationships

## Abstract

A strong emphasis is consistently placed upon the relational basis of experience within social scientific dementia research. Within this research corpus, the concept of couplehood is increasingly employed, albeit in rather undefined and loosely theorised ways. Moreover, the evaluation highlights that couplehood is often defined by a normative position that seeks to convey an affirmative perspective on dementia and spousal relationships. The lack of theoretical foundation, however, weakens the explanatory potential of the concept, both for theorising dementia, as well as for empirical research. This article critically evaluates the utility of the couplehood concept by delineating three underlying theoretical conceptions: phenomenological, interactional and relational perspectives. It will be argued that those theoretical threads offer different analytical angles and research opportunities. More thorough ontological development, however, can guide understandings of the complexities that underpin the relational experience of dementia. This will promote a conceptual starting point that offers a more balanced and multifaceted accommodation of two persons and their relationship.

## 1. Introduction

People with dementia and their family members often contend with substantial practical and emotional difficulties. This is because dementia is not only a terminal condition but also a disease that impacts memory, communication and behaviour and thus affects the full person in their contextual settings [1]. Since the dynamics and reciprocal basis of the relationship are likely to be altered by the multiple challenges engendered by the condition [2] it is vital to understand how dementia influences the nature of relationships and how relationships shape the experience of dementia. In empirical research, various forms of relationships have been addressed; a key emphasis, however, has been placed upon the experience of couples, i.e., the relationship between the person with dementia and their spouse. Taking the empirical context of the experiences of dementia into account, research approaches require a theoretical foundation that (a) does not reduce the effects of the condition to its biomedical components and takes the full person into consideration; (b) situates the person in their relational embedding.

Seeking to address those requirements, many studies have been drawing on Tom Kitwood’s influential model of personhood [3]. Personhood highlights that the experience of dementia is strongly shaped by relationships and social conditions. A person with dementia should thereby not be reduced to the neurodegenerative impacts of the disease since positive relational and environmental conditions can help to sustain personhood. Despite its success, critiques of Kitwood’s model suggest his model remains individualised and does not fully account for the bidirectional basis of relationships [4,5]. Kitwood’s model of personhood still asserts that the ‘person comes first’ [3]. This does not provide an optimal conceptual basis for a genuinely relational elucidation of experience [6]. Partly in response to those potential shortcomings of personhood, the concept of couplehood sets out to address how couples experience dementia together [7]. Rather than the person coming first, the couple becomes the primary focus of enquiry. Although the concept could be applied to other close relationships, the spousal couple is almost exclusively discussed and can function as an example for other dyadic relations. Moreover, the spousal relationship is most often characterized by particularly relevant aspects, such as shared history, cohabitation, established caring roles or the interrelation of emotional and material entanglement. While those aspects can also be found in other types of close relationships, the spousal couple can be seen as the ideal type, being therefore well suited for theoretical analysis.

Early formulations of the couplehood concept [8] built upon the relationship-focused work of Keady and Nolan [9] that addressed the ways that couples work together in response to a diagnosis of dementia. Couplehood seeks to embed the interdependencies of relationships at the centre of dementia research. The intention is not to marginalise the importance of personhood or overlook the uniqueness of individuals, but to recognise that (for those in spousal relationships) couplehood is equally important to obtain a full understanding of experience.

The couplehood concept has become increasingly applied within social scientific dementia research that includes spousal couples, being principally associated with dyadic qualitative studies [10,11]. While the concept of couplehood has thus been drawn upon extensively within dementia studies, very little focused attention has been granted to its conceptual development, which is often reduced to its normative stance. Arguing that using the concept allows a more thorough engagement with people’s experiences requires a strong theoretical foundation that moves beyond embracing the relationship—or couplehood—as simply good. This paper thus addresses the question: how can couplehood be theorised effectively, in order to represent spousal relationships that are directly affected by the experience of dementia?

Building on the premise that for the concept to be useful, it needs to capture the person in all dimensions and within their relational embedding, we interrogate the applications of the concept in research practices. Considering that theoretical insights can only be gained if couplehood is not only an aggregation of two perspectives, we argue that its theoretical depth remains underexplored [12]. In the following, we will draw on examples from the social scientific literature to delineate broad theoretical strategies for using the concept. Tearing out the ways in which a concept can be theoretically grasped will allow a better understanding of its potential and applicability. Based on this delineation, we will formulate guidelines for a more ontologically theorised conceptualisation of couplehood. In the final sections, we reflect on the possible effects of the different theoretical foundations for both the meaning of the concept itself and its usefulness for dementia research in general.

## 2. Theoretical Foundations of the Application of Couplehood

Scoping the various studies in which couplehood has been employed as a guiding concept, we identify three—theoretically highly distinct—usages. First, couplehood can be understood from a largely phenomenological perspective, through which people experience couplehood. Second, couplehood can be fathomed from a practice stance, in conducting couplehood. Third, couplehood can be read as an emerging outcome, separate from the two people forming the couple. Importantly, in empirical research, one usage cannot be aligned solely with one perspective. Rather, authors apply the concept loosely to different aspects of their research. Separating and delineating the theoretical threads, however, are essential for a more grounded understanding of the concept’s potential.

For conceptual clarity we will formulate the main perspectives described in each subsection with a shortened notation, using the letters A (signifying the person with dementia), B (signifying the partner/carer) and C (signifying couplehood). This simplified notation should allow an even clearer distinction between the different usages of the concept.

### 2.1. Experiencing Couplehood

In this first theoretical conceptualization, couplehood is used as something that is rather passively experienced or perceived. Drawing largely on phenomenological perspectives the guiding imperative is that the experience of an event or a situation (in couple settings) can better be captured through the perspective of couplehood. Koren focuses in her studies on the particular feelings generated through a new partnership in older age, which she calls “second couplehood” [13,14]. Couplehood, characterised as a shared experience is also the focus of Kim and Mee Kim’s studies on biographical disruption [15]. Wadham et al. [16] see this shared experience as a potential for the preservation of relational bonds and Albert et al. refer to the experience, perception or sense of couplehood and how it can be generated through talk about intimacy [17]. In this subjectivist perspective, different, potentially conflictual roles such as the emergence of a carer/cared-for dyad can have an impact on individuals’ perceptions of couplehood [18]. Moreover, for close relationships (such as exemplified by the spousal couple) the symptoms of dementia can be particularly challenging. A person’s changing levels of empathy, expressions of denial or personality changes strongly affect the experience of any relationship, let alone those situated within long shared histories. Importantly, when these studies focus on experiences, they entail crucial epistemological questions. How is the experience as a couple perceived? How does a couple perceive the diagnoses or effects of dementia?

Social epistemology addresses questions of how knowledge is produced and organised collectively by agents embedded within particular social relations [19]. This entails an investigation of how we could best capture people’s experiences. Within couplehood studies, there is a tendency to positively emphasise a sense of togetherness, mutuality and positivity in association with dementia and spousal relationships. Couplehood is inherently good [16]. This reflects an academic concern to challenge excessively negative cultural representations of the condition and assert the ability of people to live well with dementia [20]. Couplehood can epistemologically be aligned with an affirmative perspective and a desire to move beyond a stress/burden theoretical model and explore the ways couples generate nurturing relational contexts that can support people with dementia [8,21].

While Swall et al. acknowledge frustration and guilt within respondents’ accounts [22], the final emphasis is on an assertion of how relationships are full of rewarding experiences. Gallagher and Rickenbach recognise both positive and negative changes in perceptions of relationships [23]. Nevertheless, couplehood is aligned with a ‘positive mindset’ employed by carers as they seek to sustain an optimistic outlook and face their spouse’s condition together as a team. Molyneaux et al. also account for the divergence of perspective and disagreements between members of the couple; this is presented positively as an ability to challenge one another, which is preferable to acquiescing to avoid conflict [11].

This rather positively charged, normative notion of couplehood also impacts on the phenomenological translations of experiences and perceptions into identity, with unintended problematic consequences: Kaplan addresses the perspectives of spouses on their relationships when their partner had entered institutional care, drawing upon a ‘We’ to ‘I’ typology, from the highest “Til death do us part” to the lowest “Unmarried Marrieds” [7]. In most of these studies exploring varying levels of an ‘us’ identity [22,24], normative judgements underlie the analytical focus. Daley et al. distinguish couples defined by a We/Us grouping, and couples defined by an I/Me grouping [25]. They proceed to assert that caregivers in the We/Us grouping express more positive aspects of caring than those in the I/Me category. The notion that relationships can be defined by a positive/negative typology is also reflected in Gallagher and Beard where a small sample is divided and analysed with reference to high couplehood (high relationship closeness/satisfaction) and low couplehood (low relationship closeness/satisfaction) [26]. In addition, to explore how couples maintain a sense of continuity, a quantitative study drawing upon the couplehood concept uses a survey tool to measure positive aspects of caring [27].

Adopting methods that seek to measure levels of relational togetherness and satisfaction could offer valuable insights into relationships; however, it can be strongly queried whether a focus on people’s perceptions amounts to a sufficient utilisation and elucidation of ‘couplehood’. While the extent to which a couple defines itself as a ‘we’ could be a very key dimension of their relationship, which is amenable to meaningful analysis [28], couplehood studies that employ a graded approach to ‘We/I’ tend to present a reductive representation of relationships. These studies promulgate the sense that there is a simple linear scale from ‘I’ to ‘We’, with there being a negative association between these terms, i.e., as one dimension (we) increases, the other (I) decreases as a corollary (and vice versa). Reifying complex experiences under ‘high’ or ‘low’ couplehood typology appears to promulgate a basic hierarchical dichotomy of relationships. Articles that adopt a graded approach to couplehood thus lapse into a binary model whereby those categorised by We/Us merely need their strengths reinforced, whereas those with an I/Me orientation require help to increase their relationship satisfaction [25]. In addition, the use of ‘high’ and ‘low’ as categories has (unwitting) moral associations: ‘high’ is associated with goodness and purity, with the associations of ‘low’ offering a direct contrast.

Summarising this theoretical foundation, both the person with dementia (A) and their partner and/or carer (B) are in a somewhat passive position, experiencing or perceiving couplehood (C). This could be illustrated as follows:

A ← C

B ← C

This immediately shows, that, due to their phenomenological, subjectivist basis, these approaches manoeuvre within a rather individualist perspective. Building individual identities which are then formulated as binary I/Me or We/Us identities is related to individual perceptions and experiences. However, due to the phenomenological foundation, couplehood in these usages is understood simply in epistemological terms. How is something perceived and expressed? Additionally, perceptions can be more easily altered in order to challenge widespread negative portrayals. However, by doing that, couplehood loses its explanatory potential and largely becomes a focus of epistemological engagement and language games, ignoring people’s active roles within the constitution of the couple itself.

### 2.2. Doing Couplehood

While the first theoretical underpinning could be placed within phenomenological accounts, the second foundation lies within practice theories and interactionist approaches. Broadly speaking, couplehood here can be understood as something actively produced, both through language and practices. Language use thereby is not reduced to epistemological considerations but is seen as a tool within interactionist settings in which couplehood is negotiated. The emphasis is shifted towards the active role in producing couplehood as Hellström et al. observe, that under specific circumstances people “no longer defined themselves as part of a couple” [21] (404-5). Couplehood is something negotiated, sometimes described as mutual agreement or a rekindling of the relationship and something displayed to others [29,30,31]. Meaning-making stems from interactions within the couple [12]. When Stefansdottir et al. discuss processes of maintaining or letting go of couplehood [32], it becomes clear that in this understanding couplehood is contingent, depending on the individuals’ behaviours and (inter)actions. Methodologically, however, the interactionist approaches produce challenges in that, despite the prevalent employment of joint interviews, the direct analysis of interaction via conversational data is very marginal in couplehood research. Molyneaux et al. and Hyden and Nilsson do address conversational exchanges [11,28]; however, across a substantial majority of studies, data tend to be presented in an individualised format, with individual perspectives disaggregated from the interactional basis of the interview. As the overriding emphasis of couplehood research is upon relational dynamics, it seems something of a collective oversight for so little attention to be granted to the direct exploration of interaction.

Additionally, clearly, actions extend beyond language and communication. Drawing on interactionist perspectives, the focus of several studies lies in day-to-day actions that only produce relational contexts. In line with “doing family” approaches [18,33], which emphasise the interactional production and construction of what is called family, couplehood is identified in couples doing things together [8,34] or expressing teamwork [10]. Eskola et al. [35], for example, show how practices of intimacy can support the continuity of couplehood under the impression of dementia and Hellström et al. interrogate couplehood with regard to a model that addresses the extent to which people in couples work together or separately [21], see also [9].

In a simplified way, the theoretical conception in which individuals actively produce couplehood could be noted as follows:

A → C

B → C

Extending this still-individualised notion to interactionist approaches, this would look like this:

(A + B) → C

Couplehood is formed through the actions and interactions of A and B and is thus continuously produced and reproduced in social practice. The interactive production of couplehood is at the same time a display to the couple themselves and the outside relations [29]. This audience, including other family members, friends, acquaintances and, importantly, in the case of dementia, health care and social care personnel, is another factor in the active production of the couple.

However, two risks are associated with this foundation. First, the interactionist focus reduces couplehood to the behaviours of individuals and thus risks conflation. In particular, approaches drawing on qualitative research in the form of ‘conventional storytelling’ can impute excessive significance to human agency and the interpersonal environment [36]. This means that personal strategies conveyed by respondents tend to be captured and conveyed in a decontextualised manner. Contextual factors are addressed to some extent within couplehood literature; for example, Bielsten et al. examine the influence of neighbourhood and wider relationships [34], while Sinclair et al. seek to disentangle individual, relational and external influences upon couples’ decision-making processes [37]. Nevertheless, the overriding tendency is to focus on the micro-situation while the influence of structural and cultural components upon the immediate locale of the couple remains underexplored. A decontextualised emphasis means that couplehood does not facilitate the disentangling of moral pressures upon respective members of the couple and how these are combined via their interactions [38].

Second, the practice focus suggests that couplehood is inherently and fundamentally linked to action and interaction. When couples stop “doing couplehood” the latter disappears. In their study of spouses’ experience when their partner was living in institutional care, Førsund et al. found that while participants’ identification with couplehood fluctuated, a sense of we-ness (derived in part from an institutional status such as marriage) could be sustained even though the couple is no longer overtly working together [39]. This, therefore, recognises that there is more to an ontology of spousal relationships than a sliding scale of doing togetherness.

These risks raise the question of whether the existence of couplehood is reducible to two individuals and their direct actions and interactions. Couplehood could end up as a hermeneutic entity without context and status without sustaining properties.

### 2.3. Emerging Couplehood

The third theoretical foundation moves beyond the interactionist reduction to individuals’ actions in awarding some form of independent status or substance to couples and couplehood itself. Giving more attention to the relation as such, these perspectives could be loosely aligned with relational sociology of different variations [40].

Several perspectives that describe the process of losing couplehood or preserving couplehood [41,42] assign couplehood an essence that can be gained or lost and which is thus not reducible to the individuals the couple is comprised of. This somewhat external status of couplehood (external to both parties) can then be the specific focus of an investigation. McIntyre and Reynolds study the impact of a person’s struggles or falls on people’s relationships by granting the latter a status, somewhat independent from the individuals themselves [43]. Hellstrom et al. [44], drawing on an interactionist perspective of doing gender, look at the consequences of the possibility of preserving couplehood. Hill et al. discuss programmes and interventions that should specifically empower the relationship and the couplehood [45] and Conway et al. emphasise the role of shared resilience for the possibility to continue couplehood [46]. Moreover, external factors, such as the home, are described as being significant for preserving couplehood [42]. Couplehood itself can become a guiding compass for individuals’ actions [47]. The contributions show that couplehood is conceptualised as external to the individuals, with its own emergent properties.

Importantly, couplehood itself is not only the outcome of the interactions of A and B but can be thought of as an emergent entity, which has its own properties. In the annotation, we can use the product of A and B.

A ∗ B → C

Dementia then, it can be argued, affects A, B and C, with the latter having some independent properties and status. Discourses and other external factors similarly affect and shape A, B and C. In that sense, C is not reducible to A, and B and stands alongside the two individuals.

A + B + C

Eventually, this conceptualisation allows an independent analysis of the three components of couplehood: A, B, C. However, a potential risk of treating the relationship as a separate unit is that this conception of couplehood can overemphasise unity and understate a potential divergence of perspective within the dyad. Having a particular status for couplehood can thus lead to overstating cohesion and unity. For example, people within relationships might adopt different personal narrative strategies in response to their circumstances [48]. It is likely that these strategies will be shaped by the association that each person within the couple has with dementia, i.e., whether it is them or their partner who has the condition. This pitfall to identifying couplehood with unity and positivity is also less accommodating of a strategy of ‘political resistance’ whereby respondents might wish to convey the challenges they are encountering within their relationship in a candid manner [49]. The unified and positive nature of the relationship arguably aligns with the intention to elevate the status of the person with dementia, in response to discourses that have marginalised their voice and focused on the burdens they present to others. The unity of couplehood is not therefore predicated on dyadic balance. An oscillation between positive/negative poles and the veneration of one party within the couple (the person with dementia) means that the voice of the other person (the carer) is prone to being marginalised or silenced [50].

The unifying tendency within couplehood literature to reduce intra-couple distinctions also stems from the intention to resist the value-laden binary of ‘carer’ and ‘caregiver’. An intrinsic alignment of couples with a carer/cared-for dynamic should be avoided and some people with dementia and their partners might not identify with, or might actively resist, such a definition of their relationship. Nevertheless, it can be queried whether such a perspective should be generalised to apply to all couples on an aprioristic basis. Does this resistance to ‘carer’ or ‘caregiver’ reflect the diverse actuality of people’s relationships, or does it align more closely with academic assumptions of their best interests? Resistance to such care-related terminology arguably represents the aim to resist the negative societal perspective of dependency, whereby care recipients are positioned as passive and inferior [51]. Some couples living with dementia, however, might identify positively with terms such as carer/caregiver and feel that they represent key features of their relationship. Crucially, the endeavour to marshal the lexicon and discourage the use of certain terms could divert attention from the different ways a relationship of care can be shaped.

While the utilisation of the term couplehood indicates the intention to offer some conceptual footing, rather than simply describing the interactions of two individuals, its use often remains undertheorised. The potential of the concept extends beyond a straightforward means for labelling the referents of empirical enquiry: the addition of -hood to the term seeks to convey a status that exceeds a functional description of ‘couple’, ‘dyad’ or ‘spousal relationship’. The term ‘couplehood’ therefore notifies us of something additional and distinctive about the domain of enquiry. However, while in the perspectives sketched above, couplehood gains theoretical substance and is situated beyond the individuals that form the couple, the possibility for a clear ontological status is hardly defined.

## 3. The Way Forward: Ontological Foundations of Couplehood

Above, we have sketched three distinct theoretical foundations that could be identified in the various studies using and applying the concept of couplehood. Importantly, most of the studies do not explicitly reference these positions but implicitly draw on the very logics. Conceptual matters are often underdeveloped in those approaches, and hence we have sought to delineate the different usages. Differentiating between phenomenological, interactionist and relational foundations can help to illuminate the potential of couplehood as a theoretical term and concept. The somewhat undefined and ambiguous usage, however, also showed the necessity of a more thorough understanding of what constitutes the nature of couplehood, in particular, if it cannot be reduced to either individuals or interactions. What is it that is external to the individuals forming the couple? To what do the two members of the dyad relate? What does it refer to if we argue that couplehood itself is influenced by external factors, such as discourses? In the following, we want to sketch the ontological questions that underlie a more precise and concise formulation of the couplehood concept.

The pursuit of a social ontology is concerned with philosophical questions on the nature of social reality; for example, considering the link between human agency and social structures [52,53]. When addressing ‘couplehood’, ontological deliberations address the factors that define and comprise the basis of a spousal relationship. Couplehood imputes a metaphysical quality to the couple, which demonstrates the supra-individual basis of a sovereign relationship status. Couplehood as an ontological construct, therefore, has the scope to underscore the distinctive essence of spousal relationships. Its overarching emphasis on the couple and joint existence shows that relationships are not simply an aggregation of individuals’ experiences. This, therefore, recognises that human collectives (including dyads) have an emergent sui generis basis that cannot be reduced to individual components [54,55]. Couplehood thus potentially avoids a reductionist perspective that ascribes excessive analytical primacy to autonomous individuals.

The elevation of relational experience to a state of ‘couplehood’ takes the concept towards a sense that the couple itself is a single entity. Couplehood as an ontological concept has the potential to place emphasis on the relational entity as an object, thereby understating divergence and difference between the two subjects within the couple. As described above, couplehood is marked by both unity and difference, as are the individuals comprising the couple. An ontologically sound understanding of couplehood thus needs to avoid the mistake of a reinforcing epistemology that confuses ontological status with a normative conception of what relationships should be. Ignoring this, couplehood has the damaging potential to place a constraint upon the research corpus. For example, it could reinforce a research agenda that suppresses more negative results, which do not align with its emancipatory intentions [56]. The dialectical interplay of ontology and epistemology thereby shapes the basis of the concept: while an emphasis on couplehood avoids the notion that couples are merely an aggregate of individuals, the relation as such has no positive (or negative) substance or value.

While the intention of many couplehood approaches to reduce the distinction between people with dementia and ‘healthy others’ is laudable, this normative judgement is situated at the epistemological level and does not provide a compass for the ontological depiction of what actually constitutes the relation. Undoubtedly, language can have a distancing function: a lexicon that reduces the experience of disease and deficit positions people with dementia in dehumanising and depersonalising ways, which can be harmful [3]. A bleak and narrow prognosis can also compound feelings of disempowerment and exclusion [34]. However, the endeavour of replacing negative symbols with positive ones, shifting from a stress/burden model to ‘living well’ [20] must not alter the conceptual understanding of couplehood’s ontology.

In a similar vein, epistemology often overrides ontology in the intention to query or challenge the definition of the spouse without dementia as a ‘carer’ or ‘caregiver’ [11,27,57]. This avoids reducing the couple’s experience to the impacts of dementia and challenges the assumption that the person with dementia will be vulnerable and dependent. However, again, experience and substance cannot be reduced to the framing of positive or negative statuses: a concept needs to avoid generating a pendulum swing between these two poles which has the potential to generate a dichotomising representation of dementia, divided between the binaries of ‘tragedy’ or ‘living well’ [58]. If the aim is to depict experience more fully and accurately [20], ontological concerns need to surpass epistemological considerations. In the following, we lay out four components of an ontological approach to couplehood that could strengthen both our understanding of the experience of dementia for couples and the possibilities for empirical research drawing on the concept.

### 3.1. Capturing the Individual and the Collective

As pointed out above, couplehood as an entity must not ignore differences in analytical strata nor differences in people’s experiences. For example, a model that conflates agency and structure under a unified entity promulgates a flat, undifferentiated social unit that does not account for how unequal access to resources shapes personal experience [59]. While couples might share a very similar social vantage point, it should not be overlooked that each person in the couple is likely to have different social statuses (relating to a breadth of attributes and circumstances). Social divisions will not necessarily impact on spousal relationships in a uniform way: a potential stratification of opportunities and advantages encountered within the couple should not be marginalized [60]. A sensitive development of an ontology of couplehood thus needs to avoid downwards conflation, whereby the partial autonomy of (embodied) individual agency is subordinated to an overarching collective entity [61]. The members of the relationship contribute in varied ways to the status of the relationship.

However, how to capture both, individuality and relationality? Donati and Archer highlight how relationships should be defined by the notion of relational subjects rather than plural subjects [62]. The plural subject posits that relationships are defined by a unified ‘we think’ but does not account adequately for how this is achieved or sustained. The emphasis is on sharing the same beliefs or mutual agreement, but the mechanism for how this is achieved (and sustained over time) by two subjects remains unclear. Some couplehood usages mentioned above align with the notion of the plural subject, with a sense that the relationship is defined by a fixed mutuality. We, however, propose that the concept of ‘the relational subject’ should inform couplehood. This can provide a more balanced foundation that is amenable to capturing how personal agency is articulated within (contextualised) interactional domains. This concept underscores the intrinsically relational basis of subjectivity and accordingly does not place aprioristic precedence on the person (individualism) or the couple (holism) [63]. Relationships are treated as a complex emergent outcome of interdependency, rather than based upon a reified togetherness. “To maintain that the subject is relational means that he/she is part of a ‘We’ that is not a super-ordinate entity but is, instead, a relation” [62] (32).

There is no separation of the subject from relationships, but neither are subjectivity and agency subsumed under relational dynamics [54,62]. This can therefore capture how the actions of both persons contribute to the formation of relationship dynamics and how these dynamics (in turn) shape their ongoing actions. A and B affect C, but C also affects both A and B.

### 3.2. Continuity of Entities

By analytically treating individuals A and B as well as couplehood C as distinct entities, this also allows for a dynamic perspective, drawing on Archer’s morphogenetic approach [64]. Placing the duality of structure and agency within a morphogenetic sequence, during which structural conditioning influences social interaction, which in turn, influences structural elaboration, allows an understanding of the continuous existence of the various entities. A and B shape C through their interactions, which in turn provides the basis for the interactions of A and B. Importantly, during the morphogenetic sequence, the qualities of C can be reproduced or altered, as actors A and B can be. In other words, the constitution of couplehood changes the interactants in the process [61]. This is particularly relevant for a context in which a condition such as dementia changes A profoundly. How A is affected, though, depends (besides many other factors) on the structural conditioning of C while the changed interactions of A and B can alter (or reproduce) the qualities of C. The bidirectional dynamics that lead to the emergence of a relationship need to be recognised in order to avoid subordination to a fixed top-down construct [5].

A, B and C are constantly altered throughout the process. However, since they have their own ontological status, they do continue in their existence, despite external influences and changes.

### 3.3. External Influences Shaping A, B, C

When considering the experience of dementia, it is vital to not only address interpersonal relationships but to also consider other aspects influencing the situation. An example can be infra-agential aspects of selfhood related to embodiment [65,66]. These impacts will obviously have substantially divergent effects on each member of a couple when one of them has been diagnosed with dementia. Again, a theoretical approach that compresses dyadic experience within a unified relational concept is not optimally equipped to address this differential impact.

A sound ontological conceptualisation of couplehood as a discrete status could divert attention from this actuality. Experience is often shaped by a wider network of interpersonal relations, as well as by wider social, cultural and economic factors. Dyadic structures “no more exist in splendid isolation than individuals” [63] (13). Public discourses are one example of such external influences. A (the person with dementia) might be affected by the imperative to uphold personhood and the ideal to continue functioning within societal rules, while B (the carer) might be confronted with demands to fulfil the caring role [38]. However, additionally, C is shaped by discursive constructions, such as the expectations towards a marriage bond. In different ways, A, B and C need to be understood as distinct entities being confronted with and shaped by external influences. This in turn has effects on the morphogenetic cycles sketched above in changing and altering A, B and C.

### 3.4. Couplehood Is Not Good Per Se

Finally, such a stratified ontological approach avoids a moral charge of the couplehood concept which is sometimes criticised in Kitwood’s model of personhood where the intention is to define a moral status, rather than engage in more metaphysical considerations of identity [67]. If couplehood retains both the relational and moral orientation of the personhood term, this often includes the intention to endorse nurturing relational contexts that can support the person with dementia, with a direct focus on spousal relationships. In this context, it is noteworthy that empirical dyadic studies that do not align with, and/or actively resist, the construction of an overtly positive view, tending not to align expressly with the couplehood term [50,68,69].

In defining the relational as stratified and the ontology of the relational as an entity within the morphogenetic cycle, Donati and Archer do not treat relations as morally good in themselves [62]. On the contrary, since relations have emergent properties themselves, they can produce both relational goods and relational evils. In the context of dementia, this means that the existence of the relational entity (the couplehood) allows for positive and negative emergents. The analysis can thus focus on how a particular nature of couplehood produces positive (such as care, trust, love) or negative (such as abuse, burden, loneliness) outcomes. The analytical decision to treat couplehood as its own entity moreover allows an investigation of the effects of these relational goods on A and B, the individuals comprising the couple themselves.

## 4. Conclusions

The purpose of this article is not to dismiss the potential value of the couplehood concept or work undertaken under this ‘banner’. The focus on dyadic and relational experience offers a vital contribution to an understanding of experience for people in spousal relationships. However, as has been shown, an undertheorised conception does not contribute to a deeper understanding of a couple’s experience of dementia and can even harm the analytical potential. Delineating phenomenological, interactionist and relational accounts, we have argued for a thorough ontological foundation for the concept in order to be useful as an analytical category, as well as for empirical studies.

For empirical research, the ontological foundation of couplehood as a relational entity can contribute to a better understanding of the complexity of the effects of dementia on the person with dementia, their partner or carer and the qualities of the relationship. Experience can be grasped in its different dimensions and on different levels. The morphogenetic sequence allows an analytical distinction of different points in time, as well as of different effects and emergents. Importantly, as the analyses have shown, any relationship requires an ontological understanding and grounding. In other words, careful theorization of the spousal relationship and the effects of dementia can provide further tools for analysing other types of close relations, such as those with other family members, friends or (health) care workers.

The theoretical potential lies in strengthening the couplehood concept itself by securing it from critique as simply normative. Both members of the couple are relational subjects: the conceptual starting point is not the personhood of the person with dementia or a ‘unitary’ couple, but a more balanced and multifaceted accommodation of two people and their relationship. Couplehood is thus defined by both ‘I’ and ‘We’ statuses and their complex interrelationship—these statuses do not have a zero-sum basis. This means this enhanced model of couplehood does not start with one person and work outwards, and neither does it compress both subjects into a single relational entity. There are often two vulnerable people experiencing challenges in a care relationship, and this revised model does not prompt the researcher to ‘take sides’. In addition, it does not seek to conflate their respective experiences within a unified perspective of shared relational identity. This can guide researchers in their endeavour to grasp the complex reality of relationships, rather than setting out a preferred format for the representation of findings.

Moreover, couplehood, informed by relational subjectivity, not only focuses its analysis on relations within the dyad but also readily accommodates the influence of other relationships and wider networks. Relations could be with structural institutions and cultural norms, as well as infra-subjective phenomena associated with embodiment. This approach to couplehood and relational subjectivity is therefore compatible with a multidimensional perspective on the contextualised experience of dementia [5].

For research methodologies, this conceptualisation of couplehood entails the imperative to include the different strata in empirical research. While dyadic studies have been a prominent approach in dementia studies, they often lose sight of other strata of reality influencing the experiences of the individuals. Moreover, treating couplehood as an ontological entity might help to avoid the pitfall of selection bias, since couples in discordant relationships, or excessively challenging circumstances, will be less inclined to take part in research [56]. Couples might also seek to present themselves in a favourable manner; for example, offering accounts that portray them coping well together [70]. These are pressures upon participants and interactions that require reflexive engagement in all studies, but dyadic studies can present particular challenges, as participants are considering not only the judgements of the researcher but also the feelings of their partner [71]. Relationships are complex and difficult to define, comprising symbolic, cultural and institutional elements, as well as the (potentially divergent) perceptions of those within the relationship [54]. The theorisation of couplehood should grapple with these considerations to sharpen its concepts that can guide empirical research.

## Data Availability

Not applicable.
